# Targeting Store-Operated Calcium Entry Regulates the Inflammation-Induced Proliferation and Migration of Breast Cancer Cells

**DOI:** 10.3390/biomedicines11061637

**Published:** 2023-06-04

**Authors:** Mohammed Alqinyah, Abdullah S. Alhamed, Hajar O. Alnefaie, Mohammad M. Algahtani, Amira M. Badr, Abdullah M. Albogami, Mohamed Mohany, Yasseen A. Alassmrry, Adel F. Alghaith, Hussain N. Alhamami, Khalid Alhazzani, Ahmed Z. Alanazi, Omar Awad Alsaidan

**Affiliations:** 1 Department of Pharmacology and Toxicology, College of Pharmacy, King Saud University, Riyadh 11451, Saudi Arabia; 2 Department of Pharmaceutics, College of Pharmacy, King Saud University, Riyadh 11451, Saudi Arabia; 3 Department of Pharmaceutics, College of Pharmacy, Jouf University, Sakaka 72341, Saudi Arabia

**Keywords:** breast cancer, inflammation, TLR4, migration, proliferation, SOCE

## Abstract

Persistent challenges complicating the treatment of breast cancer remain, despite some recent undeniable successes. Sufficient evidence currently exists demonstrating the crucial role of inflammation, characterized by the enhanced activation of Toll-like receptor 4 (TLR4) and the COX-2/PGE2 pathway, in the migration and proliferation of breast cancer cells. Interestingly, the store-operated calcium entry (SOCE) pathway was shown to be essential for the TLR4 activity and COX-2 expression in immune cells such as macrophages and microglia. However, whether SOCE influences inflammatory signaling and the inflammation-induced proliferation and migration of breast cancer cells is still unknown. Thus, the current study intended to delineate the role of SOCE in the TLR4-induced inflammation, migration, and proliferation of breast cancer cells. To this end, MDA-MB-231 breast cancer cells were treated with lipopolysaccharide (LPS) to activate TLR4, BTP2 to inhibit SOCE, and Thapsigargin to induce SOCE. Following these treatments, several experiments were conducted to evaluate the proliferation and migration rates of the MDA-MB-231 cells and the expression of several inflammatory and oncogenic genes, including *COX-2*, PGE2, *IL-6*, *IL-8*, and *VEGF*. Different techniques were used to achieve the aims of this study, including qRT-PCR, Western blotting, ELISA, MTT, and wound healing assays. This study shows that SOCE inhibition using BTP2 suppressed the LPS-induced migration and proliferation of breast cancer cells. Additionally, treatment with LPS caused approximately six- and three-fold increases in *COX-2* mRNA and protein expression, respectively, compared to the controls. The LPS-induced elevations in the *COX-2* mRNA and protein levels were suppressed by BTP2 to the control levels. In addition to its effect on COX-2, BTP2 also suppressed the LPS-induced productions of PGE2, *IL-6*, *IL-8*, and *VEGF*. Conversely, SOCE induction using Thapsigargin enhanced the LPS-induced inflammation, migration, and proliferation of breast cancer cells. Collectively, these results provide evidence for the potentially important role of SOCE in inflammation-induced breast cancer progression processes. Thus, we argue that the current study may provide novel targets for designing new therapeutic approaches for the treatment of breast cancer.

## 1. Introduction

Breast cancer is a major healthcare burden that affects women worldwide due to its high incidence and mortality rates [1]. Other factors, including metastasis and drug resistance, often add to the complexities and challenges of managing and treating breast cancer. Therefore, there is a necessity to find novel approaches that aid in combating this disease. 

Cancer cell invasion is a crucial step in the progression of breast cancer, which is characterized by the ability of cancer cells to invade the surrounding tissues, ultimately leading to the metastasis of breast cancer to different organs in the body [2]. This indeed worsens the prognosis of breast cancer and complicates the therapeutic approaches to treating the disease. The invasion process is regulated by several cellular mechanisms and proteins that dictate the rate of this process. One of these proteins is the Vascular Endothelial Growth Factor (VEGF), which has been previously reported to enhance the migration and invasion of breast cancer [3]. Inflammation is another factor that influences the progression of breast cancer, adding to the complexity of breast cancer therapy [4,5]. 

Copious evidence has emerged linking inflammatory signaling to the pathophysiology and progression of different types of cancers, including breast cancer [6]. An essential component of the inflammatory signaling apparatus is the Toll-like receptor (TLR) family. Toll-like receptor 4 (TLR4) is one member of this family that plays a crucial role in the innate immune system by activating various inflammatory signaling pathways, leading to the production of proinflammatory mediators [7]. The most recognized activator of TLR4 is lipopolysaccharide (LPS), which is a component of the outer membranes of Gram-negative bacteria [8]. The activation of TLR4 by LPS initiates different molecular signaling pathways, which include NF-κB, PI3K/AKT, and MAPKs, ultimately resulting in the production of proinflammatory cytokines, for example, IL-6 and IL-8 [8]. In breast cancer cells, TLR4 activation promotes the migration of these cells and induces the production of inflammatory cytokines and oncogenes such as IL-6 and VEGF [9]. Additionally, the siRNA-mediated knockdown of TLR4 suppresses the proliferation and release of the inflammatory cytokines, IL-6 and IL-8, in breast cancer cells [10]. Furthermore, TLR4 has been found to be overexpressed in breast cancer tissue and is the most highly expressed member of the TLR family in MDA-MB-231 breast cancer cells [9,10].

Another crucial inflammatory pathway that is strongly activated by TLR4 is the Cyclooxygenase-2 (COX-2)/Prostaglandin E2 (PGE2) pathway [11]. COX-2 expression is usually upregulated in multiple cancers and is often associated with poor prognoses [12,13,14]. Like TLR4, the activation of the COX-2/PGE2 pathway has been shown to enhance the migration of breast cancer cells [15]. These data demonstrate the important role of inflammation, particularly TLR4 and COX-2, in the pathophysiology of breast cancer. 

Intracellular calcium is an essential regulator of many key processes in cancer progression, including proliferation and migration [16]. The activation of G_q_-protein-coupled receptors (G_q_PCRs) promotes the phospholipase C (PLC)–inositol 1,4,5-trisphosphate (IP_3_) signaling pathway, leading to the release of calcium from the endoplasmic reticulum (ER) to the cytoplasm [17]. The depletion of calcium stores in the ER is sensed by the stromal interaction molecules (STIMs) STIM1 and STIM2, which are ER-resident proteins that mainly act as calcium sensors, resulting in their activation [18]. The activated STIM proteins directly interact with and activate calcium release-activated calcium channels (CRACs), thereby increasing calcium entry into the cells to replenish the depleted calcium stores of the ER, in a process known as store-operated calcium entry (SOCE) [18]. 

Previous studies have illustrated the vital role of SOCE in immune signaling [19]. The inhibition of the SOCE pathway results in impaired TLR4-mediated immune responses in the microglia [20] and astrocytes [21]. However, it is not known how SOCE affects the TLR4 signaling in breast cancer cells. The main objective of the current study is to delineate the role of the SOCE pathway in regulating the immune signaling in breast cancer cells and further examine whether this pathway influences the inflammation-induced proliferation and migration of breast cancer cells.

## 2. Materials and Methods

### 2.1. Cell Culture and Reagents

MDA-MB-231 and MCF-7 were utilized as models of human breast cancer and were obtained from the American Type Culture Collection (Manassas, VA, USA). The cells were cultured in a 25 cm^2^ flask with Dulbecco’s Modified Eagle’s Medium (Gibco, Thermo Fisher Scientific, Waltham, MA, USA), containing 10% fetal bovine serum (Gibco, Thermo Fisher Scientific, Waltham, MA, USA) and a 1% penicillin–streptomycin solution (Gibco, Thermo Fisher Scientific, Waltham, MA, USA). Additionally, the cells were maintained under optimal conditions at 37 °C and 5% CO_2_. The BTP2, lipopolysaccharide, and Thapsigargin were bought from MedChemExpress (Monmouth Junction, NJ, USA). Unless stated otherwise, the cells were treated with the following drug concentrations: BTP2 (1 µM), LPS (10 μg/mL), Thapsigargin (100 nM), and serum-free medium (control).

Since the objective of this study was to examine the role of SOCE in the LPS-induced signaling and actions in breast cancer cells, we needed a cell line that responds strongly to LPS to be able to detect the changes that would be induced by the SOCE-modulating agents such as BTP2 and Thapsigargin. Based on our initial assessment and testing, we concluded that the MDA-MB-231 cell line was suitable for our study, due to its clear and strong response to LPS treatment.

### 2.2. Real-Time Quantitative Reverse Transcription Polymerase Chain Reaction

Total RNA was extracted using the TRIzol-based technique (Invitrogen, Thermo Fisher Scientific, Waltham, MA, USA), following the manufacturer’s protocol. The total RNA (1 μg) was reverse transcribed into cDNA, utilizing Reverse Transcription Master Mix for a qPCR (MedChemExpress, Monmouth Junction, NJ, USA). Low ROX SYBR Green qPCR Master Mix (MedChemExpress, Monmouth Junction, NJ, USA) was used to perform the quantitative real-time polymerase chain reaction. The human primers for the studied genes were purchased from Integrated DNA Technologies (Leven, Belgium) and their forward and reverse sequences are shown in Table 1. *GAPDH*, a housekeeping gene, was used to normalize the gene expression of each studied gene. The 2^−ΔΔCT^ method was used to perform the analyses and the data were presented as fold changes in the gene expressions, relative to the untreated group.

### 2.3. Wound Healing Assay 

A scratch assay was used to assess the migration rates in the different experimental groups. The cells were grown in 6-well plates, in which a single straight-line scratch was created in each well on 80% confluent cells using a sterile 200 µL pipette tip. The cell debris and floating cells were removed via rinsing with phosphate-buffered saline and the cells were then fed with a fresh complete medium, followed by the treatment with the designated agents. At the time zero post-scratch, images were acquired and the cells were then incubated under optimal conditions. Following incubation for 24 h, images were taken again to assess the impact of the treatment on the cell migration. All the images were captured at 4X objective using an EVOS XL Core microscope (Thermo Fisher Scientific, Waltham, MA, USA). ImageJ software (Version 1.50i) (National Institutes of Health, Bethesda, MD, USA) was used to count the number of migrated cells in the scratched area.

### 2.4. Cell Proliferation Assay

The cell proliferation was assessed using a 3-(4,5-Dimethylthiazol-2-yl)-2,5-Diphenyltetrazolium Bromide (MTT) assay. In this experiment, 2.5 × 10^4^ cells were seeded on a 96-well plate and each well contained 100 µL of cell culture medium. The cells were then treated with the indicated time for each treatment, before MTT was added for 3 h at 37 °C and 5% CO_2_ in darkness. Dimethyl sulfoxide (100 µL) was subsequently added to dissolve the formed purple formazan crystals. A microplate reader (BioTek, Elx-800, Taunton, MA, USA) was used to measure the absorbance of the samples at 570 nm.

### 2.5. Western Blotting

A radioimmunoprecipitation assay lysis buffer supplemented with a protease inhibitor cocktail (Santa Cruz Biotechnology, Dallas, TX, USA) was used to extract the whole-cell lysate proteins. The protein concentration for each protein extract was quantified using a Pierce bicinchoninic acid protein assay kit (Thermo Scientific, Waltham, MA, USA). The whole-cell lysate protein (20 g) was run and separated on a 10% SDS-polyacrylamide gel (10% SDS-PAGE) using gel electrophoresis. The proteins were then transferred into a polyvinylidene difluoride (PVDF) membrane (Merck Millipore, Darmstadt, Germany) using electrophoresis. Nonspecific protein bindings were reduced using two hours of incubation with bovine serum albumin dissolved in 1X Tween Tris-buffered saline (TBST), then the membrane was washed several times with TBST. Following this, the membrane was incubated overnight with rabbit polyclonal antibodies against human COX-2 protein (ABclonal, Woburn, MA, USA) at a concentration of 1:4000 in the refrigerator (4 °C) under constant shaking. The membrane was then incubated for one hour with horseradish peroxidase goat anti-rabbit IgG antibody (1:10,000). Western blotting luminal reagent (Santa Cruz Biotechnology, Dallas, TX, USA) was utilized to locate the protein bands over the membrane using incubation for five minutes in darkness. The Odyssey Imaging System (LI-COR, Lincoln, NE, USA) was used to capture the images and Image Studio Lite (LI-COR, USA) was used to quantify the protein expressions. The GAPDH protein (36 kDa) was utilized as a housekeeping protein. 

### 2.6. Enzyme-Linked Immunosorbent Assay

A prostaglandin E2 enzyme-linked immunosorbent assay kit (Abcam, Cambridge, UK) was used to quantify the amount of PGE2 in the cell culture medium. The cell culture medium was collected from each experimental group following the treatment for an appropriate time. The collected cell culture medium was then centrifuged for 10 min at 1500 rpm and 4 °C. The rest of the assay steps were performed according to the manufacturer’s protocol.

### 2.7. Statistical Analysis 

All the statistical analyses were performed using GraphPad Prism 9 Software (San Diego, CA, USA). The differences between the two groups were analyzed using Student’s two-tailed unpaired *t*-tests, whereas one-way ANOVA tests followed by post hoc Tukey’s tests were used to compare more than two experimental groups. A *p*-value of less than 0.05 was considered to be statistically significant.

## 3. Results

### 3.1. LPS Treatment Upregulated the Gene Expression of COX-2 and IL-6 in MDA-MB-231 but Not in MCF-7 Cells

**Figure 1:**
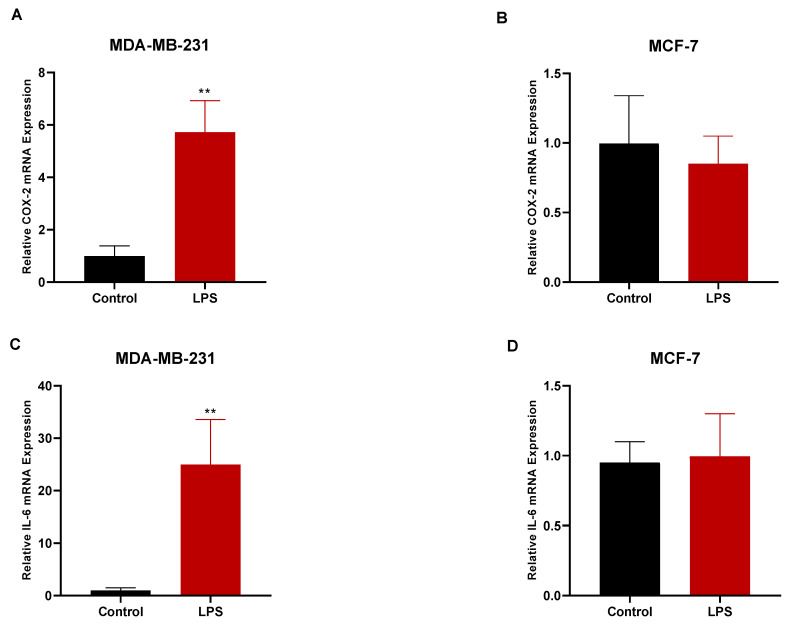
The effect of LPS treatment on the mRNA expressions of *COX-2* and *IL-6* in MDA-MB-231 and MCF-7 cells. MDA-MB-231 (**A**) and MCF-7 (**B**) cells were either untreated (control) or treated with LPS (10 μg/mL) for 48 h and *COX-2* mRNA expression in both cell lines was assessed using RT-qPCR. MDA-MB-231 (**C**) and MCF-7 (**D**) cells were either untreated (control) or treated with LPS for 4 h and *IL-6* mRNA expression in both cell lines was assessed using RT-qPCR. Expression of both *COX-2* and *IL-6* mRNAs was normalized to the housekeeping gene *GAPDH*, and relative expression was calculated using the 2^−ΔΔCt^ method. Data are presented as mean ± SEM. Student’s two-tailed unpaired *t*-test was used to determine statistical differences, where ** indicates a statistical significance relative to control. ** *p* < 0.01.

To determine the degree to which the breast cancer cell lines MCF-7 and MDA-MB-231 responded to the LPS treatment, we treated these cells with LPS and measured the expressions of the *COX-2* and *IL-6* genes in both cell lines. The LPS treatment had an evident effect on the MDA-MB-231 cells, as it significantly enhanced the production of both *IL-6* and *COX-2* mRNAs (Figure 1A,C). However, unlike the MDA-MB-231 cells, MCF-7 did not similarly respond to the LPS, as the results showed a lack of response of these inflammatory genes to the LPS treatment (Figure 1B,D). Therefore, MDA-MB-231 was chosen as an appropriate model to conduct this study. 

### 3.2. SOCE Inhibition Blocked LPS-Induced Activation of the COX-2/PGE2 Pathway

**Figure 2:**
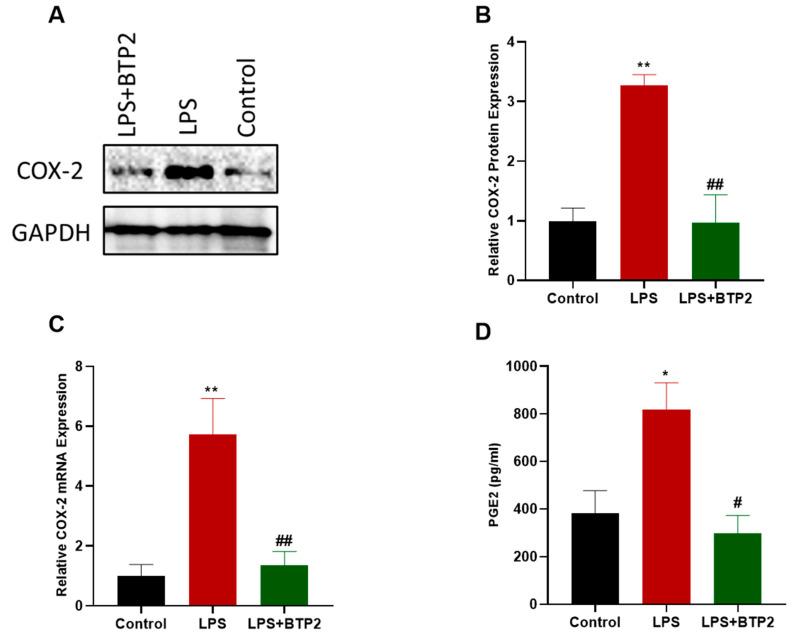
Assessing the LPS-induced activation of the COX-2/PGE2 pathway following SOCE inhibition. MDA-MB-231 cells were either untreated (control), treated with LPS alone (10 μg/mL), or treated with both LPS (10 μg/mL) and BTP2 (1 µM) for 24 h and COX-2 protein expression was measured using Western blotting. GAPDH was used as a loading control. Representative immunoblot images of COX-2 and GAPDH (**A**) are displayed. (**B**) Densitometry quantification of the band intensities in which the values of band intensities for the protein of interest (COX-2) were normalized to the values of band intensities for corresponding loading control (GAPDH). (**C**) MDA-MB-231 cells were either untreated (control), treated with LPS alone, or treated with both LPS and BTP2 for 48 h and *COX-2* mRNA expression was measured using RT-qPCR. *COX-2* mRNA was normalized to the housekeeping gene control *GAPDH* and relative expression was calculated using the 2^−ΔΔCt^ method. (**D**) MDA-MB-231 cells were either untreated (control), treated with LPS alone, or treated with both LPS and BTP2 for 24 h before the cell culture medium was collected and PGE2 levels were measured using ELISA assay. Conversion of raw absorbance values to picograms per milliliter concentration was conducted using a standard curve, following the manufacturer’s protocol. Data are presented as mean ± SEM. A one-way ANOVA test followed by post hoc Tukey’s test was used to compare different experimental groups, where * and # indicate a statistical significance relative to control and LPS, respectively. ^#^ or * *p* < 0.05 and ^##^ or ** *p*< 0.01.

To evaluate the effect of SOCE inhibition on the LPS-induced activation of the COX-2/PGE2 pathway, we measured the mRNA expression of *COX-2*, as well as the protein expressions of both COX-2 and PGE2 in the MDA-MB-231 cells, following the treatment with LPS alone or in combination with the SOCE inhibitor, BTP2. An RT-qPCR, Western blotting, and an enzyme-linked immunosorbent assay (ELISA) were used to measure the mRNA levels of *COX-2*, the protein expression of COX-2, and the PGE2 production, respectively. Our results indicate that LPS significantly enhanced the protein and mRNA levels of COX-2 in MDA-MB-231 (Figure 2A–C). More importantly, the BTP2 treatment suppressed the LPS-induced gene production of *COX-2* (Figure 2B). Similar results were observed at the protein level, as BTP2 inhibited the LPS-induced production of the COX-2 protein (Figure 2A,B). Additionally, the amount of PGE2 protein released from the cells treated with a combination of LPS and BTP2 was lower than the amount of PGE2 released from the cells treated with the LPS alone (Figure 2D), indicating that BTP2 also suppressed the LPS-induced PGE2 production in the MDA-MB-231 cells. 

### 3.3. SOCE Inhibition Suppressed LPS-Induced Inflammatory Gene Production in MDA-MB-231 Cells

**Figure 3:**
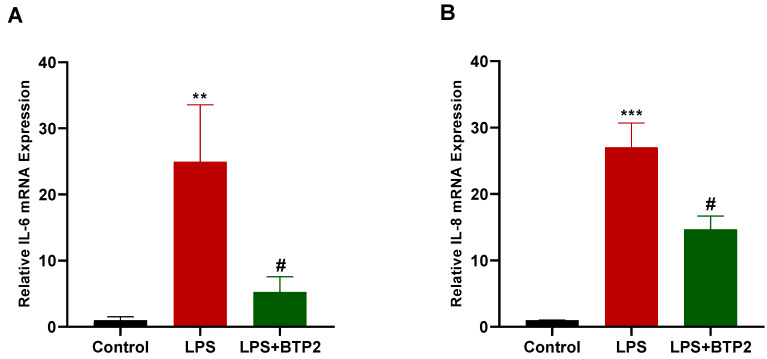
The effect of SOCE inhibition on LPS-induced gene production of *IL-6* and *IL-8*. (**A**) MDA-MB-231 cells were either untreated (control), treated with LPS alone (10 μg/mL), or treated with both LPS (10 μg/mL) and BTP2 (1 µM) for 4 h, and *IL-6* mRNA expression was measured using RT-qPCR. (**B**) MDA-MB-231 cells were either untreated (control), treated with LPS alone, or treated with both LPS and BTP2 for 48 h, and *IL-8* mRNA expression was measured using RT-qPCR. Expression of both *IL-6* and *IL-8* mRNAs was normalized to the housekeeping gene *GAPDH* and relative expression was calculated using the 2^−ΔΔCt^ method. Data are presented as mean ± SEM. A one-way ANOVA test followed by post hoc Tukey’s test was used to compare different experimental groups, where * and # indicate a statistical significance relative to control and LPS, respectively. ^#^ *p* < 0.05, ** *p* < 0.01, and *** *p* < 0.001.

To further assess the anti-inflammatory effect of BTP2 on the breast cancer cells, we examined whether the LPS-induced upregulation of the inflammatory cytokines *IL-6* and *IL-8* in the MDA-MB-231 cells would be affected by the BTP2 treatment. To this end, the MDA-MB-231 cells were treated with LPS with and without BTP2. Following these treatments, an RT-qPCR was conducted to measure the mRNA levels of *IL-6* and *IL-8* in the MDA-MB-231 cells. The data showed that the LPS treatment upregulated the mRNA expressions of both *IL-6* and IL-8, and this upregulation was significantly inhibited by the BTP2 treatment (Figure 3A,B). 

### 3.4. LPS-Induced Migration and Proliferation of MDA-MB-231 Cells Were Suppressed by SOCE Inhibition

**Figure 4:**
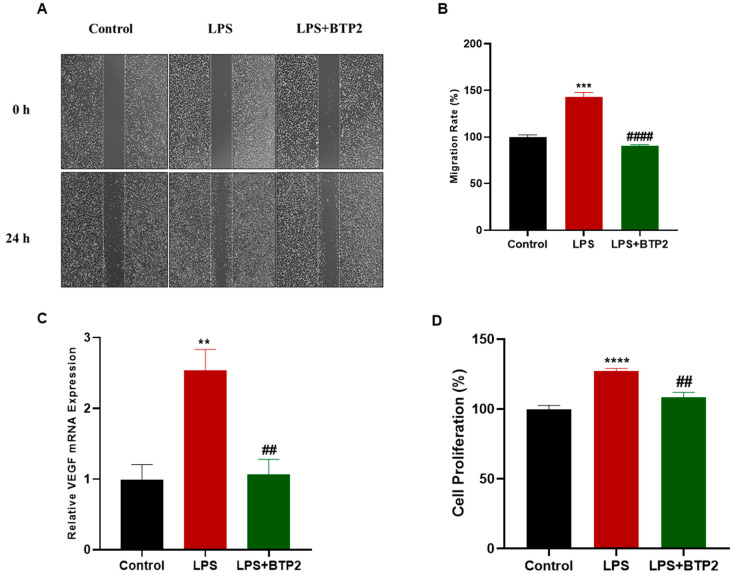
The effect of SOCE inhibition on LPS-induced migration, *VEGF* production, and proliferation. A wound healing assay was performed on MDA-MB-231 cells that were either untreated (control), treated with LPS alone (10 μg/mL), or treated with both LPS (10 μg/mL) and BTP2 (1 µM) for 24 h. Representative images of the scratch (**A**) and a graph representing the migration rate (%) (**B**) are displayed at time zero and 24 h post-treatment. (**C**) MDA-MB-231 cells were either untreated (control), treated with LPS alone, or treated with both LPS and BTP2 for 4 h before the mRNA expression of *VEGF* was measured using RT-qPCR. *VEGF* mRNA was normalized to the housekeeping gene *GAPDH* and relative expression was calculated using the 2^−ΔΔCt^ method. (**D**) MTT assay was performed to measure cell proliferation after MDA-MB-231 cells were either untreated (control), treated with LPS alone, or treated with both LPS and BTP2 for 48 h. Data are presented as mean ± SEM. A one-way ANOVA test followed by post hoc Tukey’s test was used to compare different experimental groups, where * and # indicate a statistical significance relative to control and LPS, respectively. ^##^ or ** *p* < 0.01, *** *p* < 0.001, and ^####^ or **** *p* < 0.0001.

We then intended to determine whether BTP2 would also inhibit the LPS-induced migration and proliferation of the MDA-MB-231 cells. A scratch assay was conducted to monitor the migration of the MDA-MB-231 cells following the treatments with LPS alone or in combination with the BTP2 treatment. The results indicated an increase in the migration of the MDA-MB-231 cells following the LPS treatment, but the BTP2 treatment blocked the LPS-induced migration of the MDA-MB-231 cells (Figure 4A,B). Next, we sought to determine the effects of the LPS and BTP2 treatments on the gene expression of *VEGF* using an RT-qPCR. The data showed that the LPS treatment increased the expression of the *VEGF* mRNA in the MDA-MB-231 cells and the LPS-induced upregulation of VEGF was inhibited by the BTP2 treatment (Figure 4C). Additionally, we measured the effect of LPS and BTP2 on the proliferation of the MDA-MB-231 cells using an MTT assay. The results indicated that the LPS treatment enhanced the proliferation of the MDA-MB-231 cells and the BTP2 treatment blocked the LPS-induced proliferation (Figure 4D).

### 3.5. Thapsigargin Treatment Potentiated LPS-Induced Production of Inflammatory Genes

**Figure 5:**
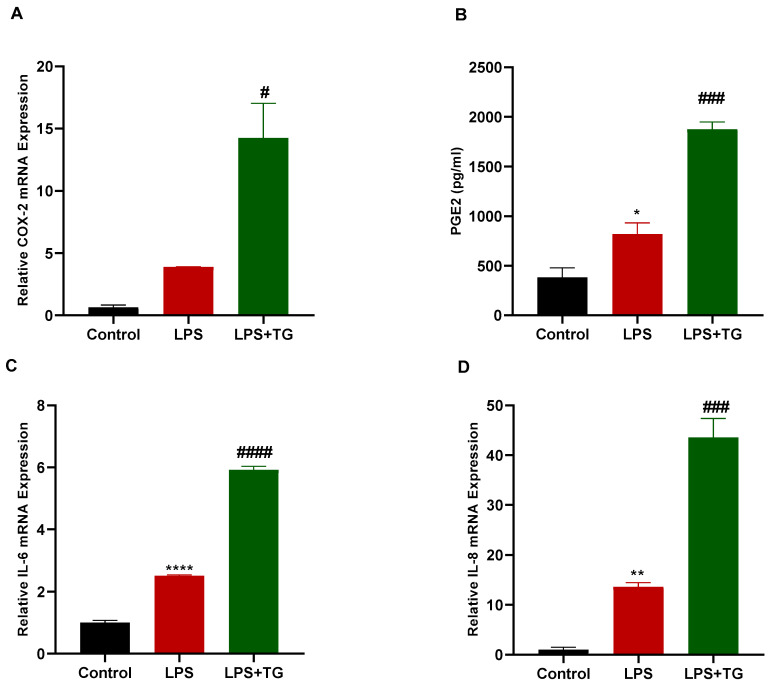
Thapsigargin effect on LPS-induced inflammation in MDA-MB-231 cells. MDA-MB-231 cells were either untreated (control), treated with LPS alone (10 μg/mL), or treated with both LPS (10 μg/mL) and Thapsigargin (100 nM) for 24 h before the mRNA expressions of *COX-2* (**A**), *IL-6* (**C**), and *IL-8* (**D**) were measured using RT-qPCR. *COX-2*, *IL-6*, and *IL-8* mRNAs were normalized to the housekeeping gene *GAPDH* and relative expression was calculated using the 2^−ΔΔCt^ method. (**B**) MDA-MB-231 cells were either untreated (control), treated with LPS alone, or treated with both LPS and Thapsigargin for 24 h before the cell culture medium was collected and PGE2 levels were measured using ELISA assay. Conversion of raw absorbance values to picograms per milliliter concentration was conducted using a standard curve following the manufacturer’s protocol. Data are presented as mean ± SEM. A one-way ANOVA test followed by post hoc Tukey’s test was used to compare different experimental groups. * and # indicate a statistical significance relative to control and LPS, respectively. ^#^ or * *p* < 0.05, ** *p* < 0.01, ^###^ *p* < 0.001, and ^####^ or **** *p* < 0.0001.

The Sarco/endoplasmic reticulum calcium ATPase (SERCA) pump inhibitor Thapsigargin is a well-established activator of SOCE in cells and has been used extensively to trigger calcium entry via the SOCE mechanism, making it a suitable tool for studying various aspects of SOCE [22]. In our study, we intended to determine whether Thapsigargin would produce the opposite effects of BTP2 on the actions of LPS in breast cancer cells. Therefore, the MDA-MB-231 cells were treated with either LPS alone or in combination with Thapsigargin. Following the treatments, the mRNA expressions of *IL-6, IL-8,* and *COX-2* were measured using an RT-qPCR, and an ELISA was used to measure the PGE2 production in the MDA-MB-231 cells. The results demonstrated that the Thapsigargin treatment significantly elevated the LPS-induced production of the *COX-2* gene, as well as the PGE2 protein, in the MDA-MB-231 cells (Figure 5A,B). In addition, the LPS-induced production of the *IL-6* and *IL-8* genes was enhanced by the Thapsigargin treatment (Figure 5C,D). 

### 3.6. Thapsigargin Enhanced the LPS-Induced Migration and Proliferation of MDA-MB-231 Cells

**Figure 6:**
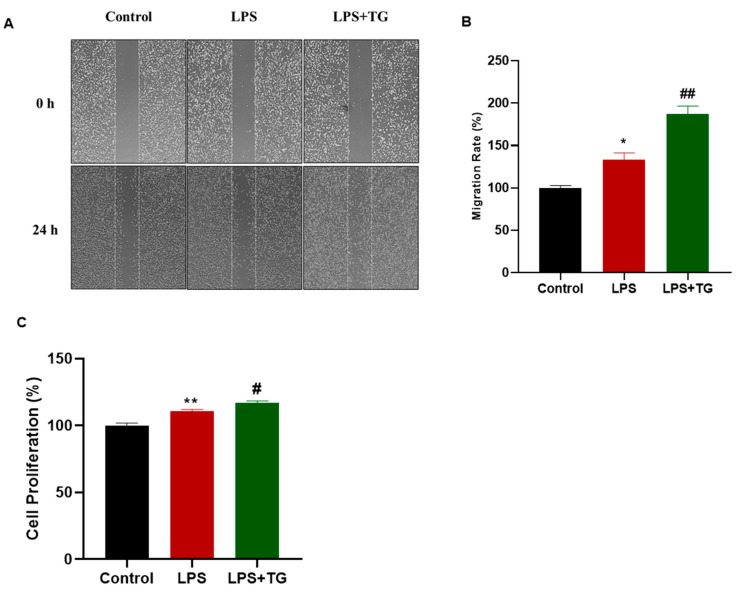
Assessment of the LPS-induced migration and proliferation of MDA-MB-231 cells following LPS and Thapsigargin treatment. A wound healing assay was performed on MDA-MB-231 cells that were either untreated (control), treated with LPS alone (10 μg/mL), or treated with both LPS (10 μg/mL) and Thapsigargin (100 nM) for 24 h. Representative images of the scratch (**A**) and a graph representing the migration rate (%) (**B**) are displayed at time zero and 24 post-treatment. (**C**) MTT assay was performed to measure cell proliferation after MDA-MB-231 cells were either untreated (control), treated with LPS alone, or treated with both LPS and Thapsigargin for 24 h. Data are presented as mean ± SEM. A one-way ANOVA test followed by post hoc Tukey’s test was used to compare different experimental groups, where * and # indicate a statistical significance relative to control and LPS, respectively. ^#^ or * *p* < 0.05 and ^##^ or ** *p* < 0.01.

We next intended to determine whether the effect of the Thapsigargin on the LPS-induced production of inflammatory genes was accompanied by an impact on the LPS-induced migration and proliferation of breast cancer cells. To achieve this aim, we repeated the scratch assay by treating the cells with Thapsigargin instead of BTP2. The results indicated that, contrary to BTP2, the Thapsigargin treatment promoted the LPS-induced migration of the MDA-MB-231 cells (Figure 6A,B). In addition, the LPS-induced proliferation of the MDA-MB-231 cells was enhanced by the Thapsigargin treatment (Figure 6C).

### 3.7. LPS Treatment Increased the Gene Expression of STIM2

**Figure 7:**
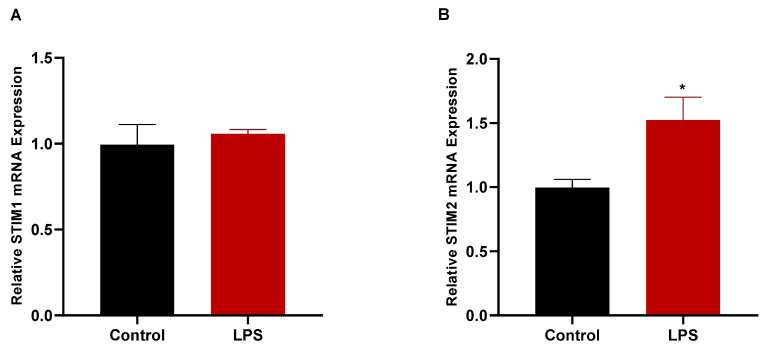
Measurement of LPS-induced changes in the gene expressions of *STIM1* and *STIM2*. MDA-MB-231 cells were either untreated (control) or treated with LPS (10 μg/mL) for 48 h before the mRNA expressions of *STIM1* (**A**) and *STIM2* (**B**) were measured using RT-qPCR. *STIM1* and *STIM2* mRNAs were normalized to the housekeeping gene *GAPDH* and relative expression was calculated using the 2^−ΔΔCt^ method. Data are presented as mean ± SEM. Student’s two-tailed unpaired *t*-test was used to determine statistical differences, where * indicates a statistical significance relative to control. * *p* < 0.05.

Throughout the study, we examined the effect of SOCE on the LPS-induced production of inflammatory genes and the cancer cell migration and proliferation. Here, we sought to determine the effect of the LPS on the expression of the SOCE-related genes *STIM1* and *STIM2*. The changes in the mRNA expressions of these genes were analyzed via an RT-qPCR following the LPS treatment of the MDA-MB-231 cells. The LPS treatment enhanced the expression of *STIM2* in the MDA-MB-231 cells (Figure 7B). However, the LPS had no significant effect on the *STIM1* gene expression (Figure 7A). 

## 4. Discussion

Breast cancer remains a challenging healthcare issue despite continuous advances in the diagnosis, prevention, and treatment of this disease. Inflammation plays several roles in the pathophysiology of breast cancer and is one of the factors that leads to cancer progression and the failure of its treatment [6]. Therefore, novel approaches designed to target specific cellular pathways to suppress the inflammatory signaling in breast cancer have the potential to become valuable tools in breast cancer therapy. Our study focused on determining whether targeting SOCE would be a successful strategy for inhibiting inflammation and the inflammation-induced proliferation and migration of breast cancer cells. 

TLR4 is a pattern recognition receptor and its activation initiates several signaling cascades that ultimately lead to the production of several pro-inflammatory mediators [7]. This receptor is potently activated by Lipopolysaccharides (LPS), which are outer membrane components of Gram-negative bacteria [8]. TLR4 activation promotes the migration and proliferation of breast cancer cells [9,10]. Previous studies have reported that some actions of TLR4 are enhanced by SOCE in other cells, such as microglia and astrocytes [20,21]. Our study herein was designed to evaluate whether the SOCE pathway can influence the TLR4 signaling in breast cancer cells and ultimately affect inflammation-induced cancer progression processes. Collectively, the findings of our study indicated that inhibiting the SOCE pathway suppressed the TLR4-induced inflammation, migration, and proliferation of breast cancer cells. Conversely, SOCE activation enhanced the TLR4 induction of these cancer processes. Thus, and to the best of our knowledge, our study is the first to report that targeting SOCE can influence the TLR4-induced migration and proliferation of breast cancer cells, as well as the production of inflammatory and cancer-related genes in these cells. 

The LPS induction of inflammatory cytokines in immune cells is well documented, as these cells highly express TLR4 and respond strongly to LPS treatment [8]. In breast cancer cells, however, there are conflicting reports regarding the degree of response of breast cancer cell lines to LPS or other inflammatory stimulants in vitro. For MDA-MB-231 cells, it has been reported that these cells display a clear inflammatory response [23,24]. However, conflicting data exist regarding the response of MCF-7 cells to inflammatory stimulants, as some studies have reported that LPS induces inflammatory responses in these cells [9,25], whereas other reports have indicated that LPS treatment or other inflammatory stimulants provoke little or no inflammatory response in MCF-7 cells compared to MDA-MB-231 cells [26,27,28]. Furthermore, there is limited information in the literature to confirm whether LPS increases the production of the inflammatory enzyme COX-2 in both the MCF-7 and MDA-MB-231 cell lines. Our data showed that LPS increased the production of *COX-2* and *IL-6* in the MDA-MB-231 cells but not in the MCF-7 cells, thereby supporting the reports that have demonstrated a poor response of MCF-7 cells to LPS treatment. This is consistent with previous findings indicating that the TLR4 expression is significantly higher in MDA-MB-231 cells in comparison to MCF-7 cells, which may explain the differences observed in the LPS responses between the two cell lines [26,29]. Due to the data reported here, LPS-treated MDA-MB-231 cells were chosen to be the model used to aid in testing the hypothesis of this research.

In MDA-MB-231 cells, the results of our study indicated that LPS stimulated the production of *IL-6*, *IL-8*, and *COX-2* mRNAs, as well as COX-2 and PGE2 proteins. Notably, the LPS-induced production of these pro-inflammatory markers was suppressed by the treatment with BTP2. These data complement previously published findings indicating that SOCE inhibition decreases the LPS-induced production of some inflammatory mediators in microglia [20] and macrophages [30], which are immune cells known for their strong responses to LPS. Additionally, it has been shown that the SOCE inhibitors, SKF-96365 and 2-APB, or the genetic knockdown of STIM1 and ORAI1 suppress the LPS-induced COX-2 production in gastric cancer cells [31]. Nonetheless, our findings showed, for the first time, that inhibiting SOCE using BTP2 can successfully suppress the TLR4-induced production of several inflammatory genes in breast cancer cells. 

The LPS treatment and TLR4 activation induced other actions in the breast cancer cells, in addition to an increase in the production of inflammatory mediators. For example, LPS promoted the migration of the MDA-MB-231 cells and increased the production of *VEGF* [9]. Additionally, the genetic knockdown of TLR4 inhibited the proliferation of the breast cancer cells [10]. This prompted us to investigate whether SOCE inhibition would also suppress the LPS-induced production of *VEGF* and the migration and proliferation of breast cancer cells. The results showed that the SOCE inhibitor BTP2 suppressed the LPS-induced migration and proliferation of breast cancer cells, as well as the LPS-induced *VEGF* production. These results further indicated that SOCE inhibition would not only suppress the TLR4-induced inflammatory signaling in breast cancer cells, but also inhibit TLR4-induced breast cancer progression processes. The data obtained in this study provide additional evidence linking SOCE to the pathophysiology of several types of cancer, including colorectal, thyroid, and breast cancers [32,33,34]. It should be noted that many other studies have focused on the role of SOCE solely in other types of cancer, whereas our study investigated the role of SOCE in the interplay between inflammation and breast cancer. Therefore, an interesting future research direction would be to expand upon the results of this study and investigate whether targeting SOCE can affect inflammation-induced cancer progression in other types of cancer, where it has been established that inflammation plays a role in their pathophysiology.

Thapsigargin is a compound that increases SOCE by inhibiting Sarco/endoplasmic reticulum calcium ATPase (SERCA), thereby causing a calcium depletion in the endoplasmic reticulum [22]. A significant number of studies have investigated the role of Thapsigargrin in several physiological and pathophysiological conditions, including cancer [35]. In immune cells, it has been shown that Thapsigargin enhances the LPS-induced production of COX-2 and other inflammatory mediators [20]. After we observed the effects of inhibiting SOCE using BTP2 on LPS-induced actions, we investigated whether SOCE activation using Thapsigargin would produce opposite effects in LPS-treated breast cancer cells. The data indicate that Thapsigargin potently enhanced the LPS-induced production of inflammatory genes in breast cancer cells, which is consistent with observations of its effects in immune cells. Thapsigargin also stimulated the LPS-induced migration and proliferation of breast cancer cells, which appears to be the opposite response to what was observed for BTP2. This result provides further evidence indicating that targeting SOCE, either by activation or inhibition, influences the TLR4-induced inflammation, proliferation, and migration of breast cancer cells. The fact that the SOCE inhibitor BTP2 suppressed the TLR4 signaling in breast cancer cells, whereas the SOCE activator Thapsigargin enhanced this signaling pathway further, suggested that the effect observed on the TLR4 signaling was mainly due to their actions on SOCE, rather than any unique non-specific actions of either drug. 

Although the focus of our research was to investigate the influence of SOCE on the LPS-mediated signaling in breast cancer cells, we also examined the effect of LPS on the expression of the SOCE-related genes *STIM1* and *STIM2*. This question was previously proposed in a few studies on immune cells and lung endothelial cells. The data obtained from these studies indicated that, in lung endothelial cells, LPS enhanced the mRNA expression of *STIM1* but not that of *STIM2* [36]. On the other hand, in microglia, LPS enhanced the protein expression of STIM2 [20]. This is interesting, since it has been suggested previously that STIM2, and not STIM1, may play a more prominent role in mediating the LPS-induced production of inflammatory mediators in macrophages [30]. The data we obtained in our study were similar to what has been observed for immune cells, as we reported that LPS induced the mRNA expression of *STIM2* but not *STIM1*. Certainly, these results are preliminary and further investigation is warranted to delineate the specific roles that each protein in the SOCE pathway play in mediating the TLR4-induced inflammation and cancer progression of breast cancer cells. This can be achieved by future studies utilizing techniques including siRNA to perform a specific knockdown of STIM1 and STIM2, which will enable researchers to identify the specific roles of these proteins in the TLR4-induced signaling of breast cancer cells. 

In addition to promoting migration and proliferation, inflammation is also involved in mediating the chemoresistance of several types of cancers, including breast cancer [37]. Interestingly, our previous study provided evidence indicating that targeting SOCE enhances cell death and suppresses inflammation following chemotherapeutic treatment in breast cancer cells [38]. This indicates that targeting SOCE may have broader applications in influencing the variety of cancer-promoting effects of the inflammatory signaling in breast cancer, such as migration, proliferation, and chemoresistance.

In conclusion, although additional investigative work is needed to further establish the specificity and efficacy of targeting SOCE in breast cancer, as well as other types of cancer, the results obtained in this study provide evidence indicating that targeting the SOCE pathway can be a potentially valuable therapeutic approach for inhibiting inflammation-induced breast cancer progression processes. 

## Figures and Tables

**Table 1:** Forward and reverse sequences of primers.

Gene Name	Forward Sequence	Reverse Sequence
*COX-2*	5′-CCCTTGGGTGTCAAAGGTAA-3′	5′-GCCCTCGCTTATGATCTGTC-3′
*IL-6*	5′-CCAGCTATGAACTCCTTCTC-3′	5′-GCTTGTTCCTCACATCTCTC-3′
*IL-8*	5′-AGCCTTCCTGATTTCTGCAG-3′	5′-GTCCACTCTCAATCACTCTCAG-3′
*VEGF*	5′-GAGGAGCAGTTACGGTCTGT-3′	5′-GTAGCTCGTGCTGGTGTT CA-3′
*STIM1*	5′-GCGGGAGGGTACTGAG-3′	5′-TCCATGTCATCCACGTCGTCA-3′
*STIM2*	5′-CCCTCACCACCCGCAACA-3′	5′-GATGTGTGGCGAGGTTAAGGC-3′
*GAPDH*	5′-GCCAAGGTCATCCATGACAACT-3′	5′-GAGGGGCCATCCACAGTCTT-3′

## Data Availability

All data that support the findings of this study are available within the article.
